# Effects of high-protein vs. high- fat snacks on appetite control, satiety, and eating initiation in healthy women

**DOI:** 10.1186/1475-2891-13-97

**Published:** 2014-09-29

**Authors:** Laura C Ortinau, Heather A Hoertel, Steve M Douglas, Heather J Leidy

**Affiliations:** Department of Nutrition & Exercise Physiology, School of Medicine, University of Missouri, 307 Gwynn Hall, Columbia, MO 65211 USA

**Keywords:** Snacking, Appetite, Satiety, Protein, Eating initiation, Energy density

## Abstract

**Background:**

The purpose of this study was to determine whether a high-protein afternoon yogurt snack improves appetite control, satiety, and reduces subsequent food intake compared to other commonly-consumed, energy dense, high-fat snacks.

**Findings:**

Twenty, healthy women (age: 27 ± 2 y; BMI: 23.4 ± 0.7 kg/m^2^) completed the randomized crossover design study which included 3, 8-h testing days comparing the following 160 kcal afternoon snacks: high-protein yogurt (14 g protein/25 g CHO/0 g fat); high-fat crackers (0 g protein/19 g CHO/9 g fat); and high-fat chocolate (2 g protein/19 g CHO/9 g fat). Participants were acclimated to each snack for 3 consecutive days. On day 4, the participants consumed a standardized breakfast and lunch; the respective snack was consumed 3-h post-lunch. Perceived hunger and fullness were assessed throughout the afternoon until dinner was voluntarily requested. An ad libitum dinner was then provided. The consumption of the yogurt snack led to greater reductions in afternoon hunger vs. chocolate (p < 0.01). No differences in afternoon fullness were detected. The yogurt snack also delayed eating initiation by approximately 30 min compared to the chocolate snack (p < 0.01) and approximately 20 min vs. crackers (p = 0.07). The yogurt snack led to approximately 100 fewer kcals consumed at dinner vs. the crackers (p = 0.08) and chocolate (p < 0.05). No other differences were detected.

**Conclusion:**

These data suggest that, when compared to high-fat snacks, eating less energy dense, high-protein snacks like yogurt improves appetite control, satiety, and reduces subsequent food intake in healthy women.

## Findings

### Background

Over the past 30 years, there has been a significant increase in the number of snacking occasions in the US, which has occurred concomitantly with the rise in obesity [1, 2]. The relationship between increased snacking and obesity may well be attributed to the types of foods typically consumed in these smaller ‘in-between meal’ eating occasions. In the US population, nearly one third of daily intake is comprised of snack foods which tend to be nutrient-poor, yet energy dense foods (i.e., desserts, salty/high fat snacks, and candy) that are high in saturated fat and/or simple sugars and may lead to energy surplus/over-eating [1, 3]. However, limited data exist regarding whether the replacement of energy dense, high-fat snacks with ‘healthier’ alternatives has a beneficial effect on food intake regulation.

Two well-established dietary factors that consistently improve appetite control, satiety, and/or reduce daily food intake include the consumption of low energy dense foods [4] and increased dietary protein [5]. Recent data from our lab demonstrated that the consumption of a less energy dense, higher protein yogurt snack led to reduced post-snack hunger, increased post-snack fullness, and delayed eating initiation compared to yogurts that were lower in protein content and higher in energy density [6]. Thus, we sought to extend the previous findings to examine whether the consumption of a less energy dense, high-protein yogurt snack leads to greater appetite control, satiety, and reductions in subsequent food intake compared to other commonly consumed snacks that are energy-dense and high in fat.

## Methods

Twenty pre-menopausal women were recruited through flyers posted on the University of Missouri campus or through the University’s email listserv. Participants were healthy, non-smoking women (age: 27 ± 2 y; BMI: 23.4 ± 0.7 kg/m^2^) who had no food allergies, eating disorders, diabetes, recent rapid weight loss/gain, not on medication that would alter appetite, and followed a typical eating pattern including 3 meals/day and an afternoon snack. All participants were informed of the study objectives, procedures, and potential risks. Written consent was obtained from all participants. The study procedures were approved by the University of Missouri’s Human Subjects Institutional Review Board.

The study incorporated a randomized, crossover design comparing three isocaloric, commonly consumed afternoon snacks (i.e., yogurt, crackers, chocolate) that varied in macronutrient composition and physical characteristics (Table 1). In general, the yogurt was less energy dense, high in protein, and low in fat, whereas the crackers and chocolate were more energy dense, low in protein, and high in fat. The participants were provided with each snack to consume, at home/work, for 3 consecutive days. On day 4 of each pattern, the participants consumed a standardized 300 kcal breakfast meal (18% protein; 61% carbohydrates; 22% fat), at home, and reported to our facility 1-h prior to their usual lunch time to begin the 8-h testing day. Each participant was placed in a comfortable room that was absent of time cues. The testing day began with the consumption of a standardized 500 kcal lunch meal (14% protein; 69% carbohydrate; 30% fat). The respective snack pattern was completed 3-h after lunch. The participants had 15 min to consume the snack and 236 mL (i.e., 8 ounces) water. Immediately following the completion of the snack, a 100 mm visual analog scale questionnaire assessing palatability was completed to assess ‘overall liking’ of the snack. In addition, computerized, 100 mm visual analog scale questionnaires assessing appetite sensations [7] were completed every 30 min throughout the afternoon until dinner was voluntarily requested. Once this occurred, the participant was fed an ad libitum dinner of pizza pockets (290 kcal/pocket; 14% protein; 63% carbohydrates; 22% fat) with 236 mL (i.e., 8 ounces) and was instructed to eat until feeling ‘comfortably full’ within 30 min. Regardless of time of dinner request, the participants were required to remain in the facility until the full 8-h testing day was completed. Water was provided ad libitum throughout the testing day. For a more detailed description of the methodology, please see refs [6, 8] which included similar experimental designs as what was incorporated within the current study.

**Table 1:** **Snack characteristics**

	Yogurt	Crackers	Chocolate
Serving	6 oz. Cup	10 Crackers	9 Pieces
Energy Content (kcal (kJ))	160 (38)	160 (38)	160 (38)
Energy Density (kcal/g)	0.94	5.10	4.87
Total Protein (g)	14	0	2
Total Carbohydrates (g)	25	19	19
Sugar (g)	20	2	18
Total Fat (g)	0	9	9
Snack Palatability (mm)	70 ± 10	80 ± 5	80 ± 10

### Data and statistical analyses

Summary statistics (sample means, net incremental area under the curve (AUC), and/or SEM) were computed for all data. A repeated measures ANOVA was applied to compare the main effects of snacking on perceived sensations, time to dinner request, and dinner energy content. When main effects were detected, post hoc analyses were performed using Least Significant Difference procedures to identify differences between treatments. All analyses were conducted using the Statistical Package for the Social Sciences (SPSS; version 21; Chicago, IL).

## Results

**Figure 1:**
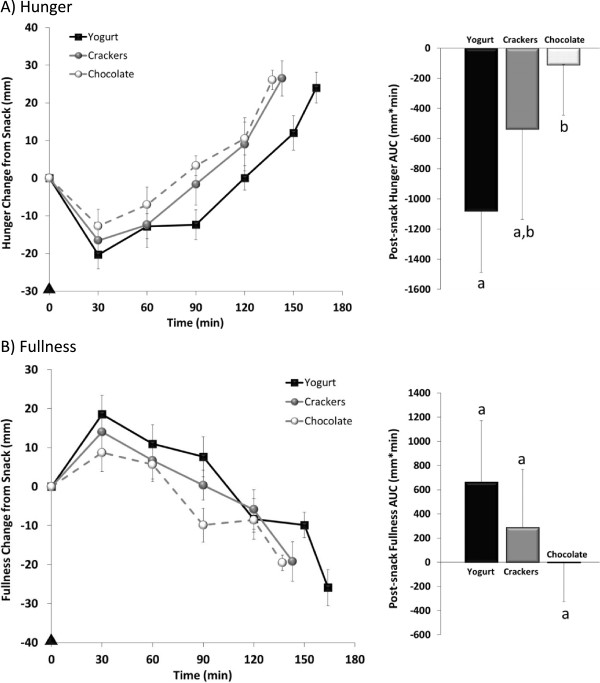
**Appetite and satiety.** Perceived hunger **(A)** and fullness **(B)** assessed from the time of snack consumption (▲) until voluntary dinner request in 20 healthy women. The post-snack net incremental area under the curve (AUC) is illustrated in the bar graphs. Data are represented as means ± SEM; Different letters denotes significance p<0.05.

Post-snack perceived hunger and fullness are shown in Figure 1. The consumption of each snack led to immediate reductions in hunger and increases in fullness, followed by gradual increases in hunger and decreases in fullness throughout the afternoon until dinner was requested. The consumption of the yogurt snack led to greater reductions in afternoon hunger AUC compared to the chocolate snack (p < 0.01). No differences in afternoon hunger AUC were detected between the yogurt vs. crackers or between the crackers vs. chocolate. In examining specific time points, the yogurt snack led to lower hunger at 90 min post-snack (33 ± 5 mm*min) compared to the chocolate (50 ± 5 mm*min, p < 0.01) and cracker (40 ± 5 mm*min, p = 0.05) snacks. No differences in afternoon fullness AUC were observed between the snacks. However, fullness at 90 min post-snack was greater following the yogurt snack (52 ± 5 mm*min) vs. chocolate (31 ± 6 mm*min, p < 0.01) but not crackers (44 ± 6 mm*min, NS). Additionally, the consumption of the crackers led to greater fullness at 90 min post-snack vs. chocolate (p < 0.03).

The consumption of the yogurt snack delayed dinner eating initiation, which is an index of satiety, by approximately 30 min compared to the chocolate snack (yogurt: 164 ± 7 min post-snack vs. chocolate: 137 ± 9 min post-snack, p < 0.01) and by approximately 20 min compared to the crackers (144 ± 10 min post-snack, p = 0.07). No differences in eating initiation were observed between the crackers and chocolate snacks.

**Figure 2:**
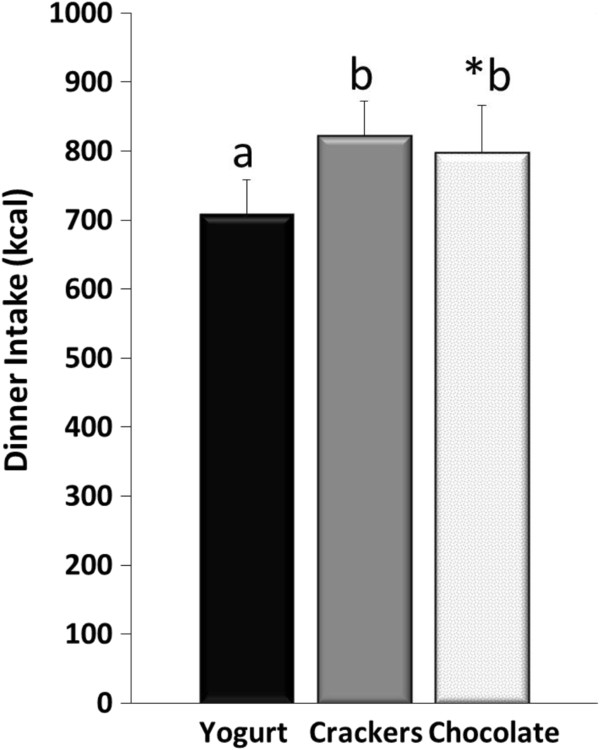
**Ad libitum dinner intake following the consumption of each the afternoon snacks in 20 healthy women.** Data are represented as means ± SEM; Different letters denotes significance p<0.05, except for *Yogurt vs. Chocolate, p=0.08.

The ad libitum dinner intake is shown in Figure 2. The consumption of the yogurt snack led to approximately 100 fewer kcals consumed at dinner compared to the crackers (p < 0.05) and chocolate (p = 0.08). No differences in dinner intake were observed between the crackers and chocolate.

## Discussion

The consumption of a less energy dense, high-protein yogurt snack led to greater reductions in afternoon hunger, delayed the onset of eating, and reduced food intake at the dinner meal compared to energy dense, high-fat snacks including crackers and/or chocolate. These data suggest that eating less energy dense, high-protein foods like yogurt improves appetite control, satiety, and reduces short-term food intake in women.

A macronutrient hierarchy exists in which the satiety effects of foods can be attributed, in part, to their nutritional composition with the consumption of dietary fat having the lowest satiety effect and protein displaying the greatest effect [9–11]. Another closely-linked dietary factor that has strong satiety properties includes the energy density of the foods [4]. Studies by Rolls et al. [4, 12, 13] consistently illustrate increased satiety and reduced food intake when consuming less energy dense foods compared to more energy dense foods. Since high-protein foods are typically less energy dense than high-fat foods, it is difficult to tease out the independent effects of macronutrient content and energy density. However, we have previously shown that, when matched for energy density, the consumption of higher protein meals lead to improved appetite control and satiety compared to normal protein versions [14].

Several previous snack studies have also examined the combined effects of reduced energy density and increased dietary protein [6, 15, 16]. Specifically, Marmonier et al. [15] included normal weight men and provided 240 kcal snacks which varied in macronutrient content and energy density. Although afternoon hunger and fullness were not different between snacks, the consumption of the low energy dense, high-protein snack delayed eating by 35 min compared to the high energy dense, high-fat snack (p < 0.05) and 25 min compared to a moderate energy dense, high-carbohydrate snack (p < 0.05). However, no difference in dinner intake was observed. In a study by Chapelot et al. [16] that included normal weight men, the consumption of a 285 kcal less energy dense, high-protein liquid yogurt snack led to a greater increase in post-snack fullness and greater decrease in post-snack hunger compared to a energy dense high-fat chocolate snack. However, eating initiation and dinner intake were not different. Lastly, in our previous study in normal weight women, we examined the effects of 160 kcal afternoon yogurt snacks, varying in energy density and macronutrient content. The less energy dense, high-protein yogurt led to reduced hunger, increased fullness, and delayed subsequent eating compared to the energy dense, high-carbohydrate version [6]. The inconsistent findings of eating initiation and diner intake between the previous studies and the current study may be attributed to the differences in snack type, macronutrient content, energy content, and energy density. However, it is important to note that, despite these differences, none of the studies showed a negative effect of consuming a less energy dense, higher protein snack in the afternoon. Further research is necessary to comprehensively identify the effects of less energy dense, protein snacks on energy intake regulation.

### Limitations

We sought to compare the satiety effects following the consumption of commercially-available, commonly consumed afternoon snacks. In using this approach, we were unable to tightly control macronutrient quantity and quality. Thus, although both high-fat snacks were similar in carbohydrate content (19 g/snack) and total fat content (9g/snack), they varied in carbohydrate and fatty acid composition. Specifically, the chocolate snack contained mostly simple carbohydrates (18 g), whereas the crackers included only 2 g of simple carbohydrates. In addition, the chocolate snack was high in saturated fat (5.5 g) and low in polyunsaturated fat (0.4 g), whereas the crackers contained only 1.5 g of saturated fat but 5.0 g of polyunsaturated fat. Some, but not all studies, have demonstrated greater reductions in hunger and/or greater increases in satiety following the consumption of complex vs. simple carbohydrates [17] and after the consumption of saturated vs. polyunsaturated fatty acids [18]. In the current study, no differences in hunger, satiety, or subsequent food intake were detected between the crackers and chocolate, suggesting that the higher saturated fatty acid content of the chocolate may have negated the negative effects of the simply carbohydrates. Collectively, these examples illustrate the need to control, not only the macronutrient content, but the type/quality as well. Other limitations include the limited assessment of only including perceived sensations of hunger and satiety in normal weight, adult women. Thus, further research incorporating both the appetitive and hormonal signals involved with energy intake regulation in overweight and/or obese individuals is warranted. Lastly, this was an acute trial over the course of a single day. Longer-term randomized controlled trials are also critical in establishing whether the daily consumption of a less energy dense, high-protein snack improves body weight management.

## Conclusion

The less energy dense, high-protein yogurt snack induced satiety and reduced subsequent food intake compared to other commonly consumed snacks, specifically energy dense, high-fat crackers and chocolate. These findings suggest that a less energy dense, high-protein afternoon snack could be an effective dietary strategy to improve appetite control and energy intake regulation in healthy women.
